# Better research by efficient sharing: evaluation of free management platforms for synthetic biology designs

**DOI:** 10.1093/synbio/ysz016

**Published:** 2019-06-20

**Authors:** Uriel Urquiza-García, Tomasz Zieliński, Andrew J Millar

**Affiliations:** 1 SynthSys and School of Biological Sciences, C. H. Waddington Building, University of Edinburgh, King’s Buildings, Edinburgh, UK; 2 Institute for Molecular Plant Sciences, D. Rutherford Building, University of Edinburgh, King’s Buildings, Edinburgh, UK

**Keywords:** synthetic biology, data management, data sharing, SBOL, JBEI-ICE, biological repositories

## Abstract

Synthetic biology aims to introduce engineering principles into biology, for example, the construction of biological devices by assembling previously-characterized, functional parts. This approach demands new resources for cataloging and sharing biological components and designs, in order to accelerate the design-build-test-learn cycle. We evaluated two free, open source software platforms for managing synthetic biology data: Joint Bioenergy Institute-Inventory of Composable Elements (JBEI-ICE) and SynBioHub. We analyzed the systems from the perspective of experimental biology research groups in academia, which seek to incorporate the repositories into their synthetic biology workflow. Here, we define the minimal requirements for a repository in this context and develop three usage scenarios, where we then examine the two platforms: (i) supporting the synthetic biology design-build-test-learn cycle, (ii) batch deposit of existing designs into the repository and (iii) discovery and reuse of designs from the repository. Our evaluation of JBEI-ICE and SynBioHub provides an insight into the current state of synthetic biology resources, might encourage their wider adoption and should guide future development to better meet the needs of this user group.

## 1. Introduction

Synthetic biology as a discipline is bringing engineering practices into biology. Practices being adopted include: standardization of components and conditions; abstraction of components and devices and decoupling of systems design and fabrication (1–5). The emergence of this approach in bioengineering has been paired with the development of registries of biological parts and devices. The community is aiming for automation of biological design, similar to the automation that has been achieved in other industries, e.g. in microelectronic designs. This requires adequate processes of abstraction and standardization, from single DNA elements to whole gene and whole circuit designs. CAD-like, assisted design and manufacturing requires access to such representations and therefore creates a need for repositories for parts and designs (6, 7).

Two free, open source platforms address the management of synthetic biology designs: JBEI-ICE and SynBioHub. The Joint Bioenergy Institute created the Inventory of Composable Elements (JBEI-ICE) (8). It is a “classical” database repository, in which each record has set of describing properties. An alternative repository is SynBioHub (https://synbiohub.org), a platform for exchanging biological designs represented in Synthetic Biology Open Language (SBOL) (9). In contrast, SynBioHub represents the metadata in a knowledge graph, utilizing the Resource Description Framework (RDF). Both resources are available as public, online services (ICE at https://public-registry.jbei.org, SynBioHub at https://synbiohub.org) or they can be set up as independent instances. Individual instances can form federations, for ICE *via* the JBEI web of registries, or for SynBioHub *via* its own web of registries or *via* SBOL stack federated queries.

The benefits of access to standardized descriptions of characterized, biological components are evident, at the level of the whole community. However, it is less clear how individual research groups gain additional benefit from using these platforms in the day-to-day, design-test-build-learn (DBTL) cycle of synthetic biology research. We believe that ICE and SynBioHub should provide additional value for individual researchers, by facilitating the DBTL cycle and helping them in their daily research, in order to encourage the wider adoption and use of the services. The virtuous circle, where the community benefits as a side-effect of these benefits to individual researchers, requires the platforms to address the motivations, requirements and user experience of all the participants. For these reasons, we evaluated how well the two systems can be integrated into typical synthetic biology workflows, from the perspective of academic research groups. We studied three scenarios:

1. a collaborative DBTL cycle,
2. the batch deposit of existing designs to the repository and
3. the discovery and reuse of designs from the repository.

### 1.1 Standpoint

The authors’ research interests comprise experimental biology, research data management, mathematical modeling and software engineering, at SynthSys, including the UK Centre for Mammalian Synthetic Biology and the Edinburgh Genome Foundry.

## 2. Materials and methods

The evaluation was primarily focused on the public online services as these are the most likely to be used by experimental biology researchers. However, standalone versions were also tested and we did not observe differences between the public, hosted and local installations, with the exception that the hosted instance of SynBioHub allows public sharing only *via* a request to the curators. The practical evaluation of public instances took place between November 2018 and February 2019, SynBioHub versions 1.3–1.4, ICE versions 5.4–5.5. The standalone instances were set up using Docker containers (same versions as public instances). The web interface was accessed using Firefox 65 on Ubuntu 18.04.2 and MacOS X Mojave.

Practical evaluation was performed by experimenting with both tools in order to achieve the desired outcomes. For each use case, we made our best efforts to discover and understand the behavior of all the software features. The strengths and limitations of each software are presented to the best of our knowledge. However, the scarcity of official documentation and/or active, ongoing development of the software could result in unintended discrepancies.

## 3. Results

### 3.1 Minimal requirements for a parts registry

We identified several features that are crucial for a registry of synthetic parts. The repositories should be able to handle information on structure, categorization and interoperability of biological parts in a coherent and accessible manner. Each repository entry can be regarded as a design definition from the abstract perspective, or as a more concrete, part record. This requires more than a simple nucleotide sequence in the repository entry, as we detail in Table 1.

**Table 1.** Minimal requirements for a parts registry (ordered by a subjective ranking of importance)

Features	SynBioHub	JBEI-ICE
Unique identifiers	ID is user defined (must be unique within collection)	ID is automatically generated (accompanied by a hidden, globally unique one)
Descriptive name	Yes	Yes
Storing exact sequence	Yes	Yes
Access permissions	Reading: public, private; Writing: private or shared with individuals	Reading and Writing: public, private, groups, individuals
Visualize sequence map	SBOL glyphs for functions, navigation to individual parts for the exact sequence	Embedded Open Vector Editor
Import common sequence formats	GenBank, FASTA, SBOL	GenBank, FASTA, SBOL
Attributions	Only one creator	Only one PI and one creator
User labels and tags	As SBOL annotations	As keywords
Functional categorization (e.g. promoter, tag)	Yes, records can be annotated using Sequence Ontology	Yes, record can be annotated with *ad hoc* keywords, sequence features can have types
Device level categorization (e.g. logic gate)	Partially, leveraging SBOL annotations	Partially, by *ad hoc* keywords
Chassis	No	Yes, “replicates in” field
Bibliography information	Yes, automatic expansion of PubMed ID	Yes, only as text in “references”
Licence information for parts	No	Yes, in intellectual property field

**Figure 1.**
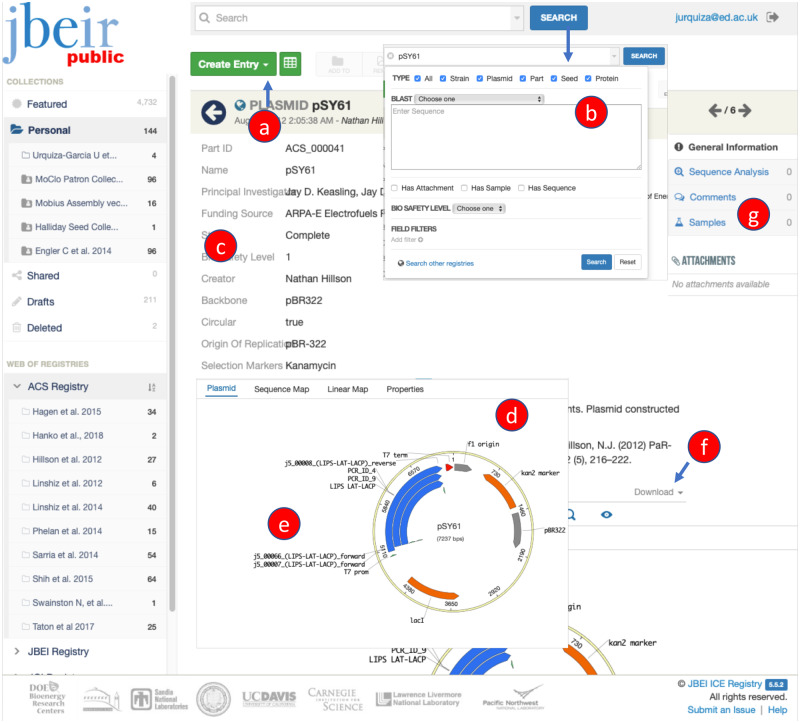
Screenshots presenting the ICE UI for ACS record #ACS_000041 (pSY61), demonstrating the main UI features: (a) design submission, (b) search, (c) metadata, (d) sequence map, (e) sequence annotations, (f) record export and (g) file attachments.

**Figure 2.**
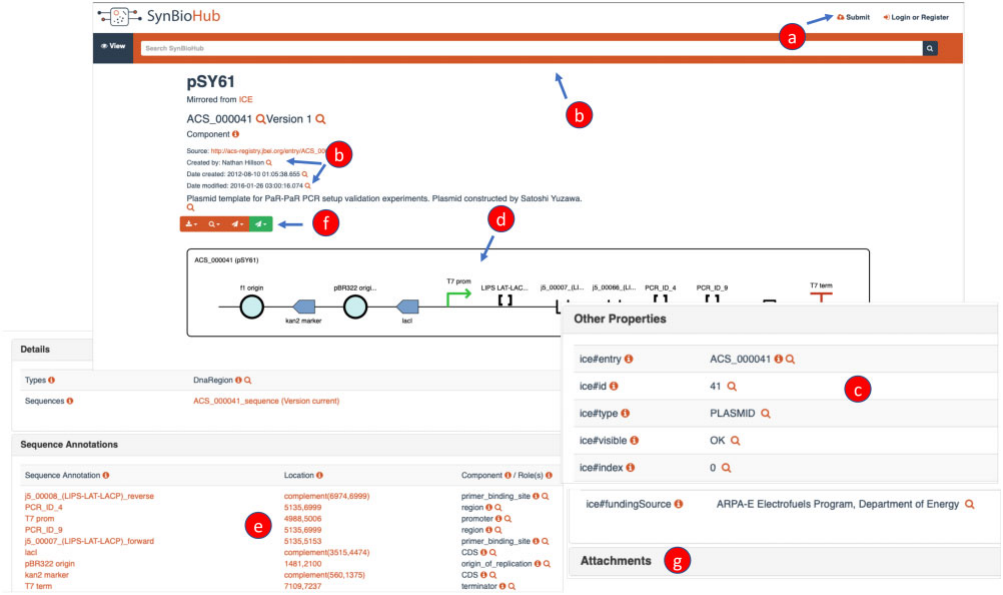
Screenshots presenting the SynBioHub UI for ACS record #ACS_000041 (pSY61), demonstrating the main UI features: (a) design submission, (b) search, (c) metadata, (d) sequence map, (e) sequence annotations, (f) record export and (g) file attachments.

Both systems cover most of these minimal requirements and their user interface (UI) is presented in Figures 1 and 2. ICE seems to be a more complete solution, as it allows online editing of part details to support incremental updates and has a rich permissions model to support collaboration. The main issue with SynBioHub is that metadata updates can only be achieved by uploading a new part-definition file (i.e. SBOL or GenBank). That involves using third party software such as the SBOLDesigner or iBioSim for handling SBOL files (10, 11). Moreover, in our experience the typical biological researcher will struggle to encode advanced metadata in an SBOL file. Surprisingly, attributions are poorly captured by both repositories, for example neither multiple authors nor identified curators are supported.

### 3.2 Scenario 1: collaborative design-build-test-learn research cycle

*Design-build phase*. The development of a new part or biological device is a multistep process involving various lab members. The design starts in an abstract form, which can include definitions of expected functions, network topology and even mathematical modeling. The current typical practice involves making “white board” abstract designs (e.g. on paper or sketched with PowerPoint). The abstract model yields parts’ specification and DNA sequences with variations, potentially creating a library of concrete instances for each abstract design. The candidate parts are researched in literature and developed in CAD-like software (Snapgene, Vector NTI, Lasergene or Benchling). The parts manufacturing starts either in the lab or is outsourced to a DNA foundry. The manufacturer can use different assembly strategies, e.g. Gibson Assembly *vs.* Type-II restriction (12–14). Each construction step should be validated by characterization data (e.g. sequencing data). Currently, the details, characterization and progress tracking often use generic systems (e.g. word processing and spreadsheet files such as Microsoft Word and Excel, organized in disk folders), with updates circulated by email. Supporting this phase extends the minimal requirements with additional features (Table 2, *design-build phase*).

**Table 2.** Requirements for collaborative DBTL research cycle

Features	SynBioHub	JBEI-ICE
Design-build phase		
Storing abstract design	Yes	No, could be mocked with dummy sequences
Sharing designs	Only with individuals, no group sharing; no read-only access	Yes, good granularity
Definition of composite parts	Yes	No, could be simulated by parent–child relationships but this means duplicating sequences annotations between records
Storing mathematical models	Yes, as attachments or external link	Yes, as attachments or external link
Assessing compatibility with assembly standards	No	No
Relating abstract design to concrete instances	Yes, if defined as “implementations” in SBOL	No, could be simulated as parent–child relationship between plasmids with dummy and real sequences
Status tracking	Possible as custom SBOL properties	Yes, field: status
Physical sample management	No	Yes
Version history	Yes	No
Confirmation sequencing data	No	Yes, chromatogram can be mapped to desired sequence
Searching/browsing by functional category	Yes	Partial, free-text search for terms
Reading commercial CAD formats	No	No
Online editing of the part record	No, only description and notes fields	Yes
Test phase		
Functional characterization (e.g. promoter activity, Km for encoded enzyme)	As free-text description or custom SBOL annotations	Only in free-text description
Link to external results	Yes, in description or notes	Yes, in experimental data section
Stable URL to link back to the part record	Yes	Yes
Key value pairs	Custom SBOL annotations	Yes
Attached files	Yes	Yes
Learn phase, discovery and research cycle		
Relationship between generations of designs	No	No
Organization of parts into collections	Yes	Yes, called folders
Participation in multiple collections	Yes	Yes
Collections tree	Yes	No
Informative table view	ID, name, description. Only for browsing, search results as cards like view	ID, name, description, status, type
API	REST and Java, SPARQL endpoint	REST and Java

SynBioHub is based in SBOL and naturally supports abstract designs and versioning of parts, whereas these features can only be partially simulated in ICE, as detailed in Table 2. On the other hand, ICE captures the concept of a biological chassis, handles physical storage of samples, and facilitates validation with sequencing data. Both platforms allow file attachments, so models or relevant data can accompany the design details. The main advantage of either repository over the “traditional approach” is that the online collections are easier to navigate and less error-prone than organizing data in disk folders. This advantage accrues most obviously when multiple members within a research group are developing many parts and is balanced against the effort required to install and learn how to use a new repository (see Discussion). Unfortunately, the relationship between abstract designs and concrete instances cannot be represented. Moreover, the discovery of suitable candidate parts is far from perfect, as we describe in the “discovery” scenario (below).

*Test phase*. Once the assembly is completed, the physical instance (DNA) must be tracked and characterized. The synthesized DNA molecules are tested for sequence correctness and their functional performance in the intended chassis. The metadata generated in this phase covers the environmental details and instrumentation used. The current practice is based on organizing sequence and data files in disk folders, with some tracking information in spreadsheets.

Whether the characterization data needs to be part of the design repository is not clear now. Storage with the part definition seems natural for some information. For example, functional characterization (e.g. promoter strength, Km for the encoded enzyme, transcription factor affinity) should be readily available from the repository. For voluminous data, for example metabolomics data in different growth conditions, a link to external resources may be a better approach. We identified desired features for both approaches (Table 2, *test phase*).

Both systems allow attaching data files to the design definitions. ICE records can be linked to external repositories, for example FAIRDOMHub or the Open Science Foundation Framework which store experimental and characterization details (15). The Experimental Data Depot (EDD) project offers even better integration as its experimental data are indexed with ICE identifiers (16). The EDD-ICE pair looks very promising, as EDD offers additional features like data visualization. However, such integration is only possible between standalone instances of ICE and EDD, not the public instance. In SynBioHub, links can be added into the free-text description or embedded in SBOL as annotations. Neither repository conveniently supports the addition of functional characterization data. They could be encoded as simple key value pairs in ICE. In the case of SynBioHub, we do not believe biologists would be able to create the required custom annotations in SBOL, even using the SBOLDesigner application (further detail is given in Section 4).

*Continuation of the research cycle.* The information gained from testing the parts and devices can be used for designing the following generation of synthetic constructs. This step will benefit from all the documentation gathered during the preceding DBTL phases. The additional features required involve tracking the relationship between new iterations of the designs, and the discovery of the existing entities (Table 2, *learn phase and research cycle*).

*Summary for the DBTL scenario.* ICE seems better suited to address the needs of a typical synthetic biology group. It might naturally be incorporated into the research workflow, as it offers clear advantages over the current practices. The adoption of SynBioHub by individual groups is more problematic. Firstly, updating records is a core, essential activity but this requires upload of an edited definition file, which is cumbersome. Secondly, biologists will not be able to provide the necessary level of details using the available SBOL editors.

### 3.3 Scenario 2: batch deposit

The affordability and speed of gene synthesis have increased considerably over the last years, for example, Twist Biosciences (https://www.twistbioscience.com) can provide thousands of 5 kb-size DNA fragments for few cents per base pair (17). This enables combinatorial approaches for part and device design, with typical libraries of hundreds of parts. Similar progress has been taking place in automatic phenotyping and high throughput assaying, which can also be outsourced to cloud providers (e.g. Transcriptic, https://www.transcriptic.com). Simultaneous handling of multiple designs becomes a necessity.

We tested the batch submission of designs to the repositories using the published Plant MoClo collection (18). The designs are stored in GenBank files and they are associated with characterization data in PDF format. We identified the following features (Table 3).

**Table 3.** Requirements for batch operations

Features	SynBioHub	JBEI-ICE
Batch designs deposition	Yes, as a definition of SBOL collection or as Zip-ed sequence files (all formats)	Yes, as Zip-ed sequence files (all formats) with a metadata file
Batch update	Yes, as a new submission with same IDs	No, however it was possible in the previous versions
Batch deposition of supporting attachments	Yes, but only linked to the collection not individual designs	Yes, if included within original deposition archive
Building records from a library definition	Yes	No
Bulk operations	Deletion and permissions setting for collection	Export, assigning to collection, permissions setting for collection

ICE supports batch upload by importing a ZIP archive file containing the part-definition files and metadata in a CSV template file. We successfully uploaded the whole MoClo collection, which the authors provided as GenBank files generated using VectorNTI software (Thermo Fisher Scientific, Waltham, MA, USA). The characterization data included in the same archive were also correctly imported into ICE. The SynBioHub can also ingest a ZIP archive, and ZIP archives of GenBank files are intended to be importable/uploadable but in practice we were unable to upload the MoClo collection directly. The upload required a prior conversion of the existing GenBank files to SBOL, which is not supported by any software in batch mode. Another option is to upload an SBOL definition of the whole collection of parts, but such an SBOL file cannot be produced by the available GUI editors. Established synthetic biology groups would be more likely to migrate their existing collections into the repositories, if the commonly used, commercial CAD formats (e.g. Snapgene, Lasergene) were also supported.

Bulk collection update is possible in SynBioHub but this is possible only by uploading new, full descriptions, not partial metadata changes (like, e.g. changing status to “tested” for selected entities). An ICE video demonstrates bulk editing using a table view but this feature was not available in the tested versions. Both systems support setting permissions for whole collections, which is natural when the individual researcher is ready to share their successful upload.

Permutations of abstract designs and parts make it easy to order hundreds of constructs from a DNA foundry, therefore we expect support for combinatorial libraries from the repositories. SBOL 2.2 introduced the representation of combinatorial genetic designs and SynBioHub implements this feature. It displays abstract designs with variable components and renders links to the associated variants. ICE does not have similar features. As way to avoid laborious preparation of hundreds of individual files for ICE batch upload, we used SBOLDesigner to expand combinatorial designs into individual files. Unfortunately, this approach also failed as ICE could not parse such files.

### 3.4 Scenario 3: discovery and reuse

A strong motivation for users using public repositories is the reuse of well-characterized parts and devices. One approach to discovery is by browsing the existing entities, which should be organized into catalogs/collections. Collections could be related not only to a publication, project, but also functional categories like promoters, plasmids, inverters or amplifiers. Each entity could belong to more than one collection, so larger repositories will require more flexible organization (Table 2, Learn phase).

SynBioHub, with nested collections, is better for organization and browsing than ICE. However, the assignment of an individual design into multiple collections, or creation of a tree of collections, cannot be done from the UI. These require an advanced SBOL file or repeated submissions of the same designs.

Synthetic biology aims for the rapid construction of devices by using a large library of parts. Finding suitable candidate parts is only possible with powerful search capabilities. Table 4 contains some exemplary test queries and how they can be executed in each system. The current search options will be insufficient for databases with thousands of candidate parts. The main reasons are the vague representation of part characterization and the free-text search, rather than a context-aware search. Discovery of parts needs to become more sophisticated as the field progresses.

**Table 4.** Examples of search use-cases

Search examples	SynBioHub	JBEI-ICE
Find by part ID, name	Yes	Yes
Find by sequence	No	Yes, BLAST
Find by author	Yes	Free text
Find by publication	No	Free text
Find by a keyword	Depending on keyword occurrence, free text search or ontology term search	Yes
Find by keyword and species	See above, plus search within species collection if defined	Free text
Find promoter	Yes	Free text
Find strong promoter for *Escherichia coli*	No. free-text search and user inspection	No. free-text search and user inspection
Find transporter for molecule X	No. Potentially search X within transporters collection if such is defined	No. As above
Find devices built with part X	No	No

Table 4 shows the search options accessible *via* the UI. As mentioned, SynBioHub utilizes the RDF representation of a knowledge graph, which can be queried using the SPARQL language. SPARQL queries are powerful and allow access to all the metadata stored about entities. In principle, all our “search scenarios” could be fulfilled with SPARQL *via* UI or the available SPARQL endpoint. Typical biologists are not able to build SPARQL queries themselves; however, SynBioHub offers a “search by example” feature, which creates a query based on the example value in a selected record (Figure 2b). This feature is of great help in learning how to construct custom queries. Unfortunately, the current SBOL editors are not user-friendly enough to create the advanced metadata descriptions that are necessary to support more advanced, semantic queries.

Another aspect of discovery and reuse is programmatic access to the repositories. Both systems provide REST and Java API for this purpose, though currently most biologists would not use these capabilities. SynBioHub additionally exposes a SPARQL endpoint (<URL>/sparql) for advanced programmatic searches.

*Installation, documentation and user experience*. Both tools were available as Docker containers and their setup with Docker was easy enough, even if maintenance and version update might represent a challenge for newcomers. Their source code can be retrieved from Git Repositories https://github.com/JBEI/ice and https://github.com/SynBioHub, however, we did not attempt to install from source. In reality, we do not expect either ICE or SynBioHub to be installed by individual groups and recommend using the existing, public instances. If individual installation is required, it should usually be centrally supported using IT expertise at the departmental/institute level.

The user documentation is acceptable for ICE. The written documentation for SynBioHub is practically nonexistent but there are some YouTube videos available. In reality, in order to discover/benefit from all the features of SynBioHub, one has to also master the definition of the SBOL standard and SBOL tools like SBOLDesigner. In both cases, we had to experiment with the repositories to discover and understand the behavior of their features. As free software and public services, neither offers dedicated customer support, however, both projects responded quickly to questions and problems raised *via* their Github issues channel.

The UIs are intuitive and generally feel well-finished, though we observed some occasional instability with the public SynBioHub, e.g. errors in file uploading. While ICE is a self-sufficient product, SynBioHub depends heavily on third party software for SBOL editing. We found the free SBOLDesigner difficult to use, which unfortunately impacts negatively on the whole user experience with SynBioHub.

## 4. Discussion

Synthetic biologists recognize the value of public data registries. However, we have not seen widespread adoption of registries in the daily work of academic research groups. The records for many of the parts, devices and associated data created by this research community remain within lab walls or in the supplementary materials of publications. This is worrying, because learning and reuse of characterized parts is essential to deliver on the promise of synthetic biology (1, 19, 20).

To be most successful, repositories should assist in the daily research workflow, rather than being only a final step of reporting during the publication process. Recording the essential information on synthetic parts incrementally, as they are developed and characterized within the lab, leads to better-quality metadata and is much less of a burden than retrieving all the (potentially scattered) elements in a laborious “big bang” of curation at the end.

Based on our evaluation, we conclude that ICE can be used on a daily basis in the synthetic biology laboratory. Obviously, the absence of a repository does not prevent the daily work of most individual synthetic biologists. The advantages for researchers come from better organization of their group’s collection of parts, where multiple lab members can update the records for individual parts (scenario 1) in the permission-controlled, shared repository, and existing libraries of parts can be quickly ingested (so long as they are present as supported sequence formats; scenario 2). Installation of a shared ICE instance should likely be a central investment for a department or research center, using IT expertise that is not reliably available in biological labs, and providing a focus for peer support by experienced users. The investment would be expected to pay off through increased productivity of the center’s researchers, greater reproducibility of their research and compliance with Open Research mandates. There are already multiple public instances of ICE registries (e.g. at Synberc, at the address noted above). The journal ACS Synthetic Biology encourages authors to submit construct designs to the ACS-ICE repository (21). The main issue with ICE is that it is registry of parts and devices but not a registry of devices made from parts, so it does not reflect the history and dependencies of device development.

SynBioHub is indeed a registry of synthetic devices made from biological parts and benefits from being constructed around the community standard SBOL. Having metadata represented as a knowledge graph is a powerful feature that could increase reuse and discovery. For example, it already allows one-click navigation to formal definitions of metadata terms. Unfortunately, this aspect is not a top priority for the biologists who produce the actual descriptions. The most relevant aspects for these, critical data producers are a frictionless deposit process and then to gain daily benefits from using the platform. To that end, SynBioHub must allow full online editing and not depend on external SBOL editors. The semantic annotation process should be biologist-friendly and hide the underlying RDF predicates. As an example of this approach, the RightField tool adds defined lists of ontology terms to Excel spreadsheets (22). The Excel templates are then reused by scientists to collect and annotate their data, without any need to understand or even be aware of the underlying ontologies.

Public repositories help to address common issues of inconsistency in naming and of data duplication, because referencing a part by its unique identifier ensures disambiguation. SynBioHub also has a new addition in SBOLExplorer, which sorts search results using the PageRank algorithm. This may help in identifying the “reference” part among similarly labeled records. Both systems support federated access, so collaborators can search, share and reference entries between different instances of ICE and SynBioHub.

While we started this evaluation assuming that ICE and SynBioHub were competing resources, we now consider them as complementary solutions with synergistic potential. The public instance of SynBioHub contains parts collections imported from the iGEM MIT Registry and from ACS-ICE. SynBioHub has the ability to create and link to entries in ICE and Benchling, benefiting for example from ICE’s handling of physical artifacts and online part editing in Benchling. We therefore imagine a mixed usage model, in which simple parts are recorded in the easier-to-use ICE and then migrated to SynBioHub as necessary, while complex devices are deposited directly in SynBioHub. We have been informed that this hybrid approach is being adopted by the Living Computing Project (https://www.programmingbiology.org).

Support for, or even integration with, commercial software for construct design (Snapgene, Vector NTI or Lasergene) would lower the existing overhead for submission to the repositories. Given the current state of synthetic biology, the repositories should prioritize support for combinatorial libraries of designs and improved batch operations, in order to attract larger deposits into the repositories. There is still missing support for assembly technologies, such as the MoClo suffix and prefix parts context. Submitting a library of devices to a DNA foundry for synthesis is a natural step in the workflow but no DNA foundry currently accepts orders in a way that would be facilitated by using these repositories (e.g. submissions of designs’ IDs). This type of integration with a CAD-like tool was previously tested at the Edinburgh Genome Foundry (23).

Both tools are open source and both have active developers who are eager to engage with the users, creating a perfect environment for the whole synthetic biology community to contribute to these platforms. There are many large scale, synthetic biology projects that could commit developer time to the shared projects. Rather than implement their own custom solution for their particular needs, they could build around ICE or SynBioHub and contribute back to the community. Another approach would be to build user-friendly interfaces to perform particular tasks (e.g. one of the complex search queries), that underneath leverage the APIs of these existing systems. Similarly, industry could engage more with both systems: commercial DNA foundries are natural candidates, as they deal with both abstract and concrete designs and have access to, or even generate, a substantial part of the metadata.

The landscape of synthetic biology software is still evolving rapidly, so our results and desired features could contribute to benchmarking or market positioning for alternative and new systems. For example, the features for sequence editing, cloning and primer design that used to be available only with standalone, commercial applications from DNAStar or SnapGene are now incorporated into electronic lab notebooks/LIMS like Benchling or TeselaGen. Combined with their inventory capabilities, this class of products form possible alternatives to ICE. As mentioned, SynBioHub already integrates with Benchling, in what we believe is a potentially fruitful direction. A strength of SynBioHub is the representation of facts as a knowledge graph, so users benefit from multiple sources that feed in new knowledge, such as from Benchling. Online resources for genetic design, in contrast, have proved challenging even for commercial providers, as Autodesk discontinued its Genetic Constructor (23) platform in 2018. However, the source code is available under the Apache 2.0 open source license, and could potentially be resurrected to create an online alternative to SBOLDesigner.

The current priority for synthetic biology is to ensure that designs are registered and documented in public repositories. Looking into the future, a further focus should be on categorization and documenting the characterization of parts and devices. The synthetic biology community is still formalizing the relevant metadata for such functional tests. One such initiative is the Minimum Information Standard for Engineering Organisms Experiments (MIEO) from The Joint Initiative for Meteorology in Biology (JIMB, http://jimb.stanford.edu). SBOL 2.3 has added a Measure class that will enable the representation of functional characterization data. SynBioHub with its intrinsic handling of RDF should easily incorporate such new description standards in future.
